# Effect of different combinations of antibiotics on fruit quality and antioxidant defense system in Huanglongbing infected Kinnow orchards

**DOI:** 10.1186/s13568-019-0871-9

**Published:** 2019-09-14

**Authors:** Sajjad Hussain, Muhammad Junaid Rao, Muhammd Akbar Anjum, Shaghef Ejaz, Ummad-ud-Din Umar, Muhammad Arif Ali, Muhammad Fasih Khalid, Muhammad Sohail, Sezai Ercisli, Muhammad Zia-Ul-Haq, Shakeel Ahmad, Syed Atif Hasan Naqvi

**Affiliations:** 1 Department of Horticulture, Faculty of Agricultural Sciences and Technology, Bahauddin Zakariya University, Multan, 60800 Pakistan; 2 Department of Plant Pathology, Faculty of Agricultural Sciences and Technology, Bahauddin Zakariya University, Multan, 60800 Pakistan; 3 Department of Soil Science, Faculty of Agricultural Sciences and Technology, Bahauddin Zakariya University, Multan, 60800 Pakistan; 4 Department of Horticulture, Faculty of Agriculture, Ataturk University, Erzurum, Turkey; 5 Office of Research, Innovation and Commercialization Lahore College for Women University, Jail Road, Lahore, Pakistan; 6 Department of Agronomy, Faculty of Agricultural Sciences and Technology, Bahauddin Zakariya University, Multan, 60800 Pakistan

**Keywords:** Antioxidant activity, Citrus greening disease, Fruit drop, Fruit yield, Fruit quality

## Abstract

Huanglongbing (HLB), also known as citrus greening disease, is the most devastating disease of citrus across the world, caused by the phloem limited fastidious bacterium ‘*Candidatus Liberibacter* spp.’. This research was conducted on HLB infected 10-year-old Kinnow orchard located at Multan, Pakistan. Different classes of antibiotics in various combinations were applied on HLB-infected trees. The antibiotic treatments were applied before flowering in February, during fruit setting in April and at fruit growth stage in June. The different antibiotics combinations used were Ampicillin sodium + Rifampicin, Cefalexin + Rifampicin, Ampicillin sodium + Cefalexin, Ampicillin sodium + Cefalexin + Rifampicin and Control (distilled water). Different fruit qualitative and quantitative attributes were examined. The application of antibiotics significantly decreased 2–11% in flower, June and pre-harvest drops as compared to control. Further, antibiotics increased fruit weight and yield by five times while the juice content, total soluble solids, ripening index, total sugars, phenolic and vitamin C content were also increased in fruits. In addition, total soluble proteins, peroxidase and catalase activities were increased in fruits harvested from antibiotic treated plants compared to control, however the superoxidase dismutase activity was decreased in fruits of antibiotic treated plants. Finally, it is concluded that application of different antibiotics combinations helps in improving the fruit yield and different quality attributes of HLB infected Kinnow trees.

## Introduction

Citrus fruits are cultivated in variable quantities worldwide in more than 140 countries with tropical or sub-tropical environments. Citrus cultivation is concentrated on both side of the equator around 35°N and 35°S (Ramana et al. 1981). Citrus fruits are well known worldwide due to their dietary fiber, vitamin C content and carbohydrates such as glucose, sucrose and fructose, which lower the cholesterol level and prevent from digestive problems. Citrus plays a vital role in human’s health and also protect from several chronic diseases (Liu et al. 2012). Citrus is susceptible to a wide range of diseases caused by fungi, bacteria, nematodes, viruses and oomycetes (Timmer et al. 2000). In most of citrus producing areas, the major disease problem is HLB (Bové 2006). HLB is caused by the unculturable, α-proteobacterium ‘*Candidatus Liberibacter*’ spp. having three causal organism but the ‘Ca. *Liberibacter asiaticus*’ (Las) the most widespread and present in Asia and America (Johnson et al. 2014; Kim et al. 2009; Koh et al. 2012) which limits movement of nutrients in the phloem ultimately affecting the tree health. The bacterium movement first starts from the insect attack sites towards root where it multiplies. Thus, the bacterium first damages the roots and then leaves (Johnson et al. 2014). After multiplication, bacteria moves upwards and destroy the phloem cells which results in blockage due to deposition of callose and proteins. The destruction of cells makes a cell wall barrier and obstructs movement of photoassimilates towards the root system (Etxeberria and Narciso 2012). This obstructed movement of photoassimilates weakens the overall root system of plant which results in reduction of plant growth, fruit yield and quality. Infected plant produces underdeveloped, misshapen, green, small fruits and aborted seeds. HLB infected fruits possess lower sugar and higher acid contents. Thus, fruits cannot even be marketed for juice purpose. The fruits do not develop color properly and remain green on the shaded side (Bové 2006; Gottwald et al. 2007; Halbert and Manjunath 2004).

In Pakistan, HLB disease causes huge losses to citrus industry especially in Sargodha and Multan districts of Punjab province. HLB disease citrus (Kinnow) fruit is small, poor quality, greenish and unmarketable and in Pakistan no proper managing strategy is available to cure the infected trees (Razi et al. 2014). Even in non-core areas of citrus in Pakistan, 40% HLB incidence was reported by Naqvi et al. (2017). To control HLB infection, there is no curative management for citrus trees (Canales et al. 2016). However, previous studies have shown that infected trees being injected with antibiotics showed reduction of HLB symptoms (Gottwald et al. 2007; Halbert and Manjunath 2004). After treating the infected scions with Ampicillin sodium at 1000 mg/L and grafting on non-infected rootstock, no bacterial titers were observed. In a previous study, different antibiotics combinations showed reduction in bacterial titers (Zhang et al. 2012). Application of antibiotics i.e., Ampicillin sodium, Sulfadimethoxine, Penicillin, Carbenicillin, Cefalexin and Rifampicin resulted in the lower number of bacterial infestations (Zhang et al. 2014). These previous findings also showed a high potential of antibiotics use for controlling/eliminating the HLB from citrus (Zhang et al. 2013; Hu et al. 2017). Antibiotics are also very much useful against other diseases i.e., fire blight which is major problem of apple and pear occurred by the pathogen *Erwinia amylovora* and destroyed the growth and production of the crop (McManus and Jones 1994). So, to date there is no possible cure of infected citrus trees are present and till now only uprooting the diseased trees and plant the new tree, is the only strategy to control HLB spread and its management which is time consuming and causes huge losses to growers. So, it is dam need to cure the infected trees and lessen the disease impact, to produce more healthy and marketable fruits. Based on this objective we have designed a research trial to cure the infected trees with different combination of antibiotics. This study will possibly reveal the role of different antibiotics combinations to improve the fruit yield and quality of HLB infected Kinnow trees. Further, the effect of antibiotics on fruit antioxidant mechanism was also investigated.

## Materials and methods

### Research area and condition

This experiment was performed on 10-years-old Mandarin trees cv. Kinnow, a hybrid of ‘King’ (*Citrus nobilis*) × ‘Willow Leaf’ (*Citrus deliciosa*), at Multan, Pakistan. Multan is located at Latitude: 30.1955600° and Longitude: 71.4752800° with arid climate having very hot summers along with mild winters. The average yearly rainfall is about 186 mm. Multan has strong sub-tropical monsoon climate, with comparatively high temperature variations, while most of the rainfalls between June and August.

### Leaves sample collection, DNA extraction and PCR protocol

HLB symptomatic leaves were collected from 50 trees for the confirmation of Las bacterium by using conventional PCR method. The symptomatic matured leaves were selected for DNA extraction from 1 to 4 twigs which contain 8 to 10 leaves. The leaf DNA was extracted by using the modified CTAB method as described by (Murray and Thompson 1980). Leaf midribs (250 mg) were grounded in liquid nitrogen to a fine powder and 2.5 ml of 2% CTAB buffer (100 mM Tris–HCl, 50 mM EDTA, 1.4 M NaCl with 2% PVP and 0.1% β-mercaptoethanol) was added in it. After that, this solution was centrifuged at 12,000 rpm for 10 min. The supernatant was collected and again centrifuged at 12,000 rpm for 10 min after adding 0.5 ml of chloroform: isoamyl alcohol (24:1). The upper phase was taken in new centrifuge tubes. Precipitation of nucleic acids were done by mixing isopropanol with equal volume and then centrifuged at 12,000 rpm for 15 min. The pellets were washed with 70% ethanol twice and then dried, and re-suspended in 100 μl of TE buffer (Dellaporta et al. 1983; Hung et al. 1999).

**Fig. 1:**
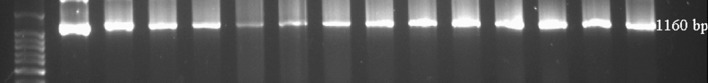
PCR product of 1160 bp amplified with primers OI1/OI2 on 1.5% agarose gel of infected Kinnow with 100 bp marker

**Fig. 2:**
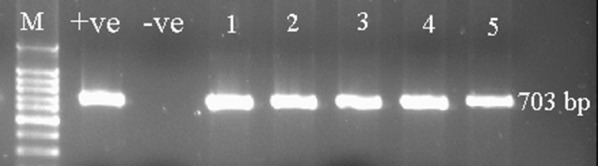
PCR product of 703 bp amplified with primers A2, J5 on 1.5% agarose gel. 1–5 from infected Kinnow leaves positive control (+ive), negative control (−ve) and M (100 bp DNA ladder)

Two sets of specific primers one from 16S rDNA region (OI1, OI2) and other from outer membrane protein gene region *rplKAJL*-*rpoBC* operon (β-operon) A2/J5 were used for the detection of *C. Librebacter* asiaticus (Jagoueix et al. 1996) (Table 1). PCR reaction was carried out in My cycler (Biorad, USA). The reaction mixture contain, 25 μl of reaction mixture was used containing 0.25 mM of each dNTPs, 2 μM of each primer, 0.2 units *Taq* DNA polymerase (Fermantas), 1x PCR buffer, 2.5 mM MgCl_2_ and 1 μl DNA template. The thermal cycle conditions for OI1 and OI2 primers were: one cycle at 94 °C temperature for 3 min; 35 cycles at 94 °C temperature for 30 s, annealing at 60 °C for 30 s and extension at 72 °C for 30 s, followed by final extension for 10 min at 72 °C. For primer A2/J5 only annealing temperature was changed to 50 °C (Jagoueix et al. 1996). The PCR product was visualized on 1.5% agarose gel (Figs. 1 and 2). The trees with positive HLB infection were selected for further study.

**Table 1:** Description of specific primers used for the amplification of *Candidatus Liberibacter*

Name	Sequence
OI1 forward primer	5-GCGCGTATGCAAGAGCGGCA-3
OI_2_ reverse primer	5-GCCTCGCGACTTCGCAACCCAT-3
A2 forward primer	5′-TATAAAGGTTGACCTTTCGAGTTT-3′
J5 reverse primer	5′-ACAAAAGCAGAAATAGCA CGAACAA-3′

#### Selection and application of treatments

A total number of 30 HLB infected trees were selected for antibiotic treatments. Randomized complete block design (RCBD) was used with five treatments and three replications while each treatment unit has 2 trees. Three different antibiotics from three classes were selected (Table 2) and four different treatment combinations were made for their application (Table 3). For control, only distilled water was used. All five treatments were applied at three different stages i.e. before flowering (February, 2015), at fruit setting (April, 2015) and at fruit growth stage (June, 2015). Moreover, before flowering and at fruit set stages, antibiotic treatments combinations were sprayed and stem injected on the same day on the same tree but at fruit growth stage antibiotic treatments were applied in the soil with higher concentration (2 g/10 l) under the canopy of the tree. Antibiotics were injected at 15–25 cm above the soil in the stem by making hole of 4–5 mm diameter wide and 3 cm long.

**Table 2:** Antibiotics, their classes and concentrations used to control HLB in infected Kinnow orchard

Antibiotics class	Chemical compound	Code	Working conc. (mg/l)
Beta-Lactam	Ampicillin sodium	Amp	1000
Cephalosporins	Cefalexin	Cef	100
Ansamycin	Rifampicin	Rif	50

**Table 3:** Combination of different antibiotics treatments applied to control HLB in infected Kinnow orchard

Treatments	Working conc. (mg/l)
Ampicillin sodium + Rifampicin	1000 + 50
Cefalexin + Rifampicin	100 + 50
Ampicillin sodium + Cefalexin	1000 + 100
Ampicillin sodium + Cefalexin + Rifampicin	1000 + 100 + 50
Control (distilled water)	–

### Parameters studied

#### Fruit set, June and pre-harvest drop (%)

For flower drops, ten randomly branches were tagged on each treated tree. All flowers were counted manually and fruit setting was observed. After this, the number of fruit set was divided by the total number of flowers of the branch and multiplied by 100. Same procedure was adopted for June fruit drop and pre-harvest fruit drop.

#### Fruit sampling and physical analysis

Twenty one fruits from all four sides of the trees were sampled and total fruit weight (g) was measured by using electronic weighing balance (Shimadzu BW-3200S, Japan) and average fruit weight (g) was calculated. For juice extraction, fruits were cut into two halves and all the juice were extracted and average juice weight (g) and content (%) was calculated. Fruit peel thickness (mm) was measured through digital vernier caliper (Vitage Helios, Germany). Further, peel and rag contents were calculated and expressed in percentage (%). Total fruit yield was calculated by weighing of all fruits from a single tree at harvesting and yield was expressed in tons per hectare.

#### Biochemical analysis of fruits

For biochemical analysis 21 fruits were harvested randomly from all four sides of the trees. The total soluble solids from the juice were determined at room temperature by using a hand refractometer. The titratable acidity was determination by following the method of Hortwitz (1960). Ripening index, the ratio of TSS (°Brix) to titratable acidity (%) of the juice was calculated as suggested by (Hardy and Sanderson 2010).

The method of (Hortwitz 1960) was followed to determine sugar contents. Juice extract (10 ml) was poured into 250 ml flasks and 25 ml solution of lead acetate (25%), 100 ml distilled water and 10 ml solution of potassium oxalate (20%) were added and final volume of 250 ml was made with distilled water. The solution was filtered and filtrate solution was used for the determination of various types of sugars.

Methanol extraction was used to extract antioxidants from juice. For this, 20 ml of juice along with 50 ml of methanol was taken in 100 ml of flask. Then flask was placed in a water bath with an automatic shaker for 2 h at room temperature. With the help of rotary evaporator extract was settled and concentrated to a volume of 10 ml for antioxidants analysis as described by (Shimada et al. 1992).

For the determination of total phenolic content (TPC), 1 ml juice was taken in centrifuge tubes and centrifuged at 4 °C for 5 min at 8000 rpm. After centrifugation, supernatant was collected and TPC was measured by the method followed by (Gorinstein et al. 2001).

Reaction mixture for total soluble proteins was prepared by taking 200 µl sample extract, 20 µl of dye and 780 µl water. The reaction mixture without sample extract was used as a blank. Absorbance was read at 595 nm and was expressed in mg/ml by using Bradford assay (Bradford 1976). Antioxidants, enzymatic activity of the juice samples was determined as, 0.5 ml juice was homogenized in 5 ml of extraction buffer (50 mM phosphate buffer, pH 7.8), followed by centrifugation at 1200 rpm for 10 min at 4 °C. The supernatant was collected and used for the study of superoxide dismutase (SOD), (Giannopolitis and Ries 1977), catalase (CAT) and peroxidase (POD) (Maehly and Chance 1955).

### Statistical analysis

Data were analyzed by using Analysis of Variance (ANOVA), and LSD test was used to compare the significant differences between treatments at 0.05% level of probability by using statistical software (Statistix 8.1).

## Results

### Effects of antibiotic treatments on fruit drop and fruit physical characteristics

Flower drop was significantly decreased among the treatments as compared to control. The maximum flower drop was observed in Control (distilled water) (83.41%), followed by Ampicillin sodium + Cefalexin (79.35%), while the minimum flower drop was noted in Ampicillin sodium + Rifampicin (73.70%) (Table 4). The June drop was maximum in control (12.02%), followed by Cefalexin + Rifampicin (10.19%), while the minimum June drop was in Ampicillin sodium + Cefalexin (8.39%). In case of pre-harvest drop, Control (distilled water) showed the maximum fruit drop (4.35%), followed by Ampicillin sodium + Cefalexin (3.25%), while the Ampicillin sodium + Rifampicin showed the minimum fruit drop (2.14%) (Table 4). The effect of different antibiotics treatments was significant in increasing fruit weight, while control treatment showed the minimum fruit weight. The highest av. weight of fruit was recorded in Ampicillin sodium + Rifampicin (149.87 g), followed by Ampicillin sodium + Cefalexin (142.87 g). The minimum av. weight of fruit was noted in control (117.13 g) (Table 4). The antibiotic treatments significantly increased fruit juice content and the highest av. juice content was observed in Ampicillin sodium + Rifampicin (44.29%), while the lowest av. juice content was observed in control (33.12%). Similarly, the highest fruit yield was observed in antibiotic treated plants and the maximum fruit yield was recorded in Ampicillin sodium + Rifampicin (19.71 tons/ha) while, the minimum was in control (3.63 tons/ha) (Table 4). To conclude, all the antibiotic treatment significantly enhanced the physical characteristics of fruits but the Ampicillin sodium + Rifampicin treatment gives the highest fruit yield, total weight and juice contents by 4–10% with least fruit drop than control.

**Table 4:** Flower and fruit drops (%) and fruit physical characteristics as affected by different antibiotic treatments

Treatments	Flower drop (%)	June drop (%)	Pre-harvest drop (%)	Av. fruit weight (g)	Av. juice content (%)	Yield (ton/ha)
Ampicillin sodium + Rifampicin	73.70 c	9.68 b	2.14 c	149.87 a	44.62 a	19.71 a
Cefalexin + Rifampicin	77.25 b	10.19 ab	2.46 bc	133.80 b	38.44 b	15.11 b
Ampicillin sodium + Cefalexin	79.35 b	8.39 b	3.25 b	142.87 ab	38.62 b	16.82 ab
Ampicillin sodium + Cefalexin + Rifampicin	74.73 c	10.07 b	2.84 bc	138.60 ab	39.69 b	14.82 b
Control (distilled water)	83.41 a	12.02 a	4.35 a	117.13 c	33.12 c	3.63 c

Means within a column with different letters are significantly different at P ≤ 0.05

### Effects of antibiotic treatments on fruit biochemical characteristics

The antibiotics treatments significantly increased reducing, non-reducing and total sugar contents in juice. The antibiotic treatment Ampicillin sodium + Rifampicin _-_showed the highest reducing (2.31%) and total sugars (5.51%) contents in juice, while the control treatment showed the lowest reducing (1.53%) and total sugars (2.59%) (Table 5). Non-reducing sugar content was the highest in Cefalexin + Rifampicin (3.25%), while the lowest was observed in control (2.59%).

**Table 5:** Fruit biochemical characteristics as affected by different antibiotic treatments

Treatments	Reducing sugars (%)	Non-reducing sugars (%)	Total sugars (%)	Antioxidant activity (%)	Antioxidant capacity (mM trolox/100 ml)	Total Phenolic content (µg GE/ml)
Ampicillin sodium + Rifampicin	2.31 a	2.96 ab	5.51 a	95.52 b	10.56 a	444.68 a
Cefalexin + Rifampicin	2.16 a	3.25 a	5.43 a	98.28 a	2.71 b	446.89 a
Ampicillin sodium + Cefalexin	1.92 ab	3.24 a	5.24 a	94.83 b	9.69 a	417.95 ab
Ampicillin sodium + Cefalexin + Rifampicin	2.15 a	2.69 b	4.88 b	95.79 ab	7.74 a	413.03 ab
Control (distilled water)	1.53 b	2.59 b	4.36 c	93.52 b	10.12 a	359.83 b

Means within a column with different letters are significantly different at P ≤ 0.05

The antibiotic treatments significantly increased antioxidant activity and capacity of fruit juice. The highest antioxidant activity was recorded in Cefalexin + Rifampicin (98.28%), while the minimum antioxidant activity was observed in control (93.52%). In case of antioxidant capacity, the maximum value was recorded in Ampicillin sodium + Rifampicin (10.56%), while the minimum value of antioxidant capacity was observed in Cefalexin + Rifampicin (2.71%). As for phenolic content, significantly higher content was recorded in Cefalexin + Rifampicin (446.89) as compared to control (359.83) which showed the lowest content (Table 5).

### Effects of antibiotic treatments on fruit physico-chemical characteristics

Antibiotic treatments significantly increased av. peel thickness as compared to control. The maximum av. peel thickness was recorded in Ampicillin sodium + Rifampicin (3.35 mm), while the minimum was noted in control (2.28 mm). The highest av. peel content was recorded in Ampicillin sodium + Rifampicin (32.34%), while low av. peel content was observed in Control (distilled water) (24.19%) (Table 6). Significantly higher av. rag content was recorded in Cefalexin + Rifampicin (24.46%) as compared to control (18.97%). In case of total soluble solids, highest value was recorded in Ampicillin sodium + Rifampicin (11.00%), while less total soluble solids were noted in control (8.83%). In contrast to total soluble solids, the highest titratable acidity was recorded in control (2.12%), while the lowest titratable acidity was observed in Ampicillin sodium + Rifampicin (0.72%). Significant increase in ripening index among the treatments was recorded in Ampicillin sodium + Rifampicin (15.25%) and Ampicillin sodium + Cefalexin (15.25%) as compared to control Control (distilled water) (4.29%) (Table 6).

**Table 6:** Fruit physico-chemical analysis as affected by different antibiotic treatments

Treatments	Peel thickness (mm)	Peel content (%)	Rag content (%)	Total soluble solids (°Brix)	Titratable acidity (%)	TSS: acidity
Ampicillin sodium + Rifampicin	3.35 a	32.34 a	21.55 ab	11.00 a	0.72 b	15.25 a
Cefalexin + Rifampicin	3.00 ab	27.87 ab	24.46 a	10.50 a	1.62 a	7.06 bc
Ampicillin sodium + Cefalexin	2.84 abc	27.62 ab	22.34 ab	10.16 a	0.74 b	15.25 a
Ampicillin sodium + Cefalexin + Rifampicin	2.57 bc	27.92 ab	19.63 b	10.83 a	0.91 b	12.03 ab
Control (distilled water)	2.28 c	24.19 b	18.97 b	8.83 b	2.12 a	4.29 c

Means within a column with different letters are significantly different at P ≤ 0.05

### Effects of antibiotic treatments on fruit soluble proteins and antioxidant enzymatic and non-enzymatic activity

Antibiotic treatments significantly increased total soluble proteins as compared to control. The highest total soluble proteins were recorded in Ampicillin sodium + Cefalexin + Rifampicin (0.73 mg/ml), while Control (distilled water) (0.26 mg/ml) showed the minimum total soluble proteins (Table 7). Superoxide dismutase activity was higher in control (58.44 min^−1^ mg protein^−1^) as compared to antibiotic treated plants, while the minimum activity was observed in Ampicillin sodium + Rifampicin (33.60 min^−1^ mg protein^−1^). The different antibiotics treatments significantly increased peroxidase and catalase activities, while control treatment showed the minimum peroxidase and catalase activities. The maximum peroxidase activity was observed in Cefalexin + Rifampicin (0.68 mmol min^−1^ mg protein^−1^) compared to control which showed the minimum peroxides activity (0.24 mmol min^−1^ mg protein^−1^). Similarly, the maximum catalase activity was noted in Ampicillin sodium + Cefalexin + Rifampicin (27.32 mmol min^−1^ mg protein^−1^) while, the minimum was observed in control (9.50 mmol min^−1^ mg protein^−1^) (Table 7). Vitamin C content was also recorded the maximum in Ampicillin sodium + Cefalexin (35.74 mg/100 ml), while the lowest was detected in Cefalexin + Rifampicin (29.36 mg/100 ml) and control (29.40 mg/100 ml) (Table 7).

**Table 7:** Fruit soluble proteins and antioxidant enzymatic and non-enzymatic activity as affected by different antibiotic treatments

Treatments	Total soluble proteins (mg/ml)	Superoxide dismutase (IU min^−1^ mg protein^−1^)	Peroxidase (IU mmol min^−1^ mg protein^−1^)	Catalase (IU mmol min^−1^ mg protein^−1^)	Vitamin C content (mg/100 ml)
Ampicillin sodium + Rifampicin	0.49 b	33.60 c	0.45 ab	18.75 b	33.77 ab
Cefalexin + Rifampicin	0.39 bc	51.60 ab	0.68 a	14.04 bc	29.36 b
Ampicillin sodium + Cefalexin	0.47 b	51.56 ab	0.47 ab	17.95 b	35.74 a
Ampicillin sodium + Cefalexin + Rifampicin	0.73 a	46.72 b	0.55 a	27.32 a	32.34 ab
Control (distilled water)	0.26 c	58.44 a	0.24 b	9.50 c	29.40 b

Means within a column with different letters are significantly different at P ≤ 0.05

## Discussion

Citrus is grown all over the world and occupies a prominent position among all other fruits. Citrus fruits are enriched in nutritional quality and well known due to their thirst suppressing property (Nagy and Attaway 1980). Citrus is a good source of vital nutrients, metabolites, dietary fibers etc. which protects humans from chronic disease even cancers and also helps in improving health due to its antioxidant capability of juice (Liu et al. 2012). Citrus plants are prone to biotic (bacteria, fungus, virus etc.) and abiotic (drought, salt, high temperature etc.) stresses; among them HLB bacterial disease is very lethal disease of citrus worldwide having no possible cure and no resistant reported to date. However, some researchers have some positive effects of antibiotic in controlling and managing the HLB infected citrus (Zhang et al. 2011, 2013). To control the HLB infection in citrus orchards, antibiotics applications were made which significantly improved yield and fruit quality of HLB infected Kinnow trees. Application of different antibiotics treatments increased fruit set (%), decreased fruit drop and, hence, a large number of fruits were harvested from HLB infected trees especially from Ampicillin sodium + Rifampicin treated ones. In a previous study on HLB infected orchard, large quantity of fruit drop was observed before harvesting (Bassanezi et al. 2009). However, in this study results showed a significant decrease in fruit drop and increase in fruit set (%). In another study, yield of HLB infected *Citrus reticulata* trees was increased after antibiotic GA_3_ treatment (Shokrollah et al. 2011). Similar results were also found by (Zhang et al. 2014), who found that ampicillin sodium were very effective in controlling HLB infection in citrus and less number of bacterial titers were observed after the treatment of ampicillin sodium. Further the fruits from HLB infected trees were usually small, distorted and inferior in quality (Mishra et al. 2011). Different classes of antibiotics have different mode of action to kill the bacteria. Some antibiotic kills and destroy large amount of bacterial population for example Ampicillin sodium 1000 mg/l application on HLB infected bud stick significantly reduces the Las bacterial population (Zhang et al. 2013, 2014) so less presence of bacteria means lower infection or less disease pressure so the host plant produce healthy fruits with better biochemical quality attributes.

In this study, greater total soluble solids and high peel thickness and juice contents (%) were obtained from Ampicillin sodium + Rifampicin and Cefalexin + Rifampicin. Moreover, higher fruit yield was achieved by antibiotic applications especially for Ampicillin sodium + Rifampicin and Ampicillin sodium + Cefalexin. Similar findings were observed when plants were treated with antibiotics and GA_3_, which showed more weight, juice (%), peel thickness, peel content and yield as compared to non-treated plants (Shokrollah et al. 2011). It was also observed that healthy or asymptomatic fruits had more weight, high juice content, less acidity and more TSS as compared to HLB infected fruits (Bassanezi et al. 2009). In the present study, high fruit weight, juice content (%) and low acidity was achieved in antibiotics treatment Ampicillin sodium + Rifampicin, while control Control (distilled water) has the highest acidity and lowest fruit weight and juice content (%). Moreover, high soluble solids were observed in antibiotics treated fruit when compared to control. In previous studies, HLB infected fruits had higher acid content, low sugars and bitterness in taste (Bové 2006). In this study, significantly higher sugar content was observed in all antibiotic treatments as compared with Control (distilled water). However, (Baldwin et al. 2009) reported that ascorbic acid and malic acids contents showed very little or no significant difference by applying antibiotics treatments.

It has been reported that, HLB infected plants show less production of defense-related pathogen-response proteins (Nwugo et al. 2013). In the present study, significantly low total soluble proteins were observed in control which means that the bacterium damaged the protein structure of cells. Increased infestation of pathogens caused increase in the activities of SOD in plants (Durner and Klessig 1995). Similarly, in our experiment, high SOD was observed in Control (distilled water) compared to antibiotic treated fruits indicating high bacterium infestation in Kinnow fruit. In contrast, the POD and CAT activities were high in fruits of antibiotic treated plants as compared to control. Saikia et al. (2004) reported that under stress condition, the defense system weakens and POD activity decreases. The CAT activity inhibition results in increase in H_2_O_2_ at cellular level and promotes cell death rate (Levine et al. 1994). Our results demonstrated that in control tress, which was in extreme stress condition, POD and CAT activities were too low, its mean that their defense system totally collapsed and it also enhanced the cell death rate. This results in overall decrease in fruit yield and quality attributes. Hence, we concluded that antibiotics treatments especially Ampicillin sodium + Rifampicin enhanced the fruit yield and quality in HLB-infected Kinnow trees. We concluded that the application of different antibiotics combinations helps in improving the fruit yield and different quality attributes of HLB infected Kinnow trees.

## Data Availability

The authors declare that all data supporting the findings of this study are available from the corresponding authors upon request.
